# Exploring absolute and relative measures of exposure to food environments in relation to dietary patterns among European adults

**DOI:** 10.1017/S1368980018003063

**Published:** 2018-12-07

**Authors:** MGM Pinho, JD Mackenbach, J-M Oppert, H Charreire, H Bárdos, H Rutter, S Compernolle, JWJ Beulens, J Brug, J Lakerveld

**Affiliations:** 1 Amsterdam UMC, Vrije Universiteit Amsterdam, Department of Epidemiology and Biostatistics, Amsterdam Public Health, De Boelelaan 1089a, Amsterdam, The Netherlands; 2 Sorbonne Université, Institute of Cardiometabolism and Nutrition, Department of Nutrition, Paris, France; 3 Equipe de Recherche en Epidémiologie Nutritionnelle (EREN), Centre de Recherche en Epidémiologie et Statistiques, Inserm (U1153), Inra (U1125), Cnam, COMUE Sorbonne Paris Cité, Université Paris 13, Bobigny, France; 4 Université Paris Est, Lab-Urba, UPEC, Créteil, France; 5 Department of Preventive Medicine, Faculty of Public Health, University of Debrecen, Debrecen, Hungary; 6 Department of Social and Policy Sciences, University of Bath, Bath, UK; 7 Department of Movement and Sport Sciences, Faculty of Medicine and Health Sciences, Ghent University, Ghent, Belgium; 8 Julius Center for Health Sciences and Primary Care, University Medical Center Utrecht, Utrecht, The Netherlands; 9 Amsterdam School of Communication Research (ASCoR), University of Amsterdam, Amsterdam, The Netherlands; 10 Faculty of Geosciences, Utrecht University, Utrecht, The Netherlands

**Keywords:** Food environment, Exposure, Relative/absolute measures, Dietary patterns, European adults

## Abstract

**Objective:**

To explore the associations of absolute and relative measures of exposure to food retailers with dietary patterns, using simpler and more complex measures.

**Design:**

Cross-sectional survey.

**Setting:**

Urban regions in Belgium, France, Hungary, the Netherlands and the UK.

**Participants:**

European adults (*n* 4942). Supermarkets and local food shops were classified as ‘food retailers providing healthier options’; fast-food/takeaway restaurants, cafés/bars and convenience/liquor stores as ‘food retailers providing less healthy options’. Simpler exposure measures used were density of healthy and density of less healthy food retailers. More complex exposure measures used were: spatial access (combination of density and proximity) to healthy and less healthy food retailers; density of healthier food retailers relative to all food retailers; and a ratio of spatial access scores to healthier and less healthy food retailers. Outcome measures were a healthy or less healthy dietary pattern derived from a principal component analysis (based on consumption of fruits, vegetables, fish, fast foods, sweets and sweetened beverages).

**Results:**

Only the highest density of less healthy food retailers was significantly associated with the less healthy dietary pattern (*β* = −129·6; 95 % CI −224·3, −34·8). None of the other absolute density measures nor any of the relative measures of exposures were associated with dietary patterns.

**Conclusions:**

More complex measures of exposure to food retailers did not produce stronger associations with dietary patterns. We had some indication that absolute and relative measures of exposure assess different aspects of the food environment. However, given the lack of significant findings, this needs to be further explored.

Abundant availability of foods in general and high accessibility to high-energy (ultra-processed) foods high in salt, sugar and/or saturated fat characterize current food environments worldwide^(^ ^1^ ^)^. Promoting healthier food environments through policy actions may contribute to healthier eating and consequently to obesity prevention at the population level^(^ ^2^ ^–^ ^4^ ^)^.

There is evidence supporting a link between the residential food environment – the distribution of food retailers within an individual’s residential area – and dietary behaviours^(^ ^5^ ^–^ ^7^ ^)^, but the evidence is inconsistent. A systematic review from Caspi *et al*. showed that the density of food retailers was significantly associated with dietary intake in thirteen out of the twenty studies reviewed. Studies using the distance to food retailers provide less consistent results: seven out of thirteen studies found no significant associations with dietary intake and two studies presented mixed results^(^ ^8^ ^)^. The review from Bivoltsis *et al.* also found that density measures produce more consistent effect sizes than distance measures; they recommended future studies to follow a multi-method approach where ideally a combination of measures is used to assess both availability and accessibility^(^ ^9^ ^)^.

The assessment of exposure to the neighbourhood food environment is complex and context-dependent^(^ ^10^ ^)^. In studies on exposure to food retailers in relation to diet, measures of geographic accessibility (i.e. proximity) and measures of geographic availability (i.e. density) are most often used^(^ ^8^ ^,^ ^11^ ^)^. A potential limitation of assessing exposure to food retailers using simpler measures (e.g. only proximity or density) is that it may not reflect the complexity of exposure to food retailers. For instance, if the distance of two study participants to their nearest food retailer is similar but the density of retailers in their neighbourhood is very different, the use of a proximity-based measure such as distance would not fully capture the difference in exposure. Therefore, while both measures may be relevant to define exposure, a combination of measures (e.g. an absolute measure that accounts for both density and proximity or a relative measure that accounts for more than one type of food retailer) may be a better option. For example, Salze *et al*. used the estimation of spatial accessibility based on a ‘potential accessibility index’ that encompasses functions of the weighted inverse distance to destinations within a specific area, as such allowing to take account of both proximity and availability^(^ ^12^ ^)^. The spatial accessibility measure was used before to explore the relation between fast-food access and body weight^(^ ^13^ ^)^.

Another potential limitation of many studies on the food environment is a focus on absolute measures of exposure, namely exposure to only one type of food retailer while ignoring the relative influence of a variety of food retailers^(^ ^14^ ^,^ ^15^ ^)^. For instance, if only density of fast-food restaurants in a neighbourhood is considered without accounting for the presence of other types of food retailers, results might be biased because access to healthier food retailers may balance the influence that less healthy food retailers have on dietary choices^(^ ^16^ ^)^. Therefore, it has been argued that the use of relative measures which take into account the variety of food retailers within the broader food environment might be preferable^(^ ^8^ ^,^ ^16^ ^–^ ^20^ ^)^. An example of a relative measure is the modified Retail Food Environment Index (mRFEI), representing the percentage of healthier food retailers relative to the total amount of food retailers in the area^(^ ^21^ ^)^. While this measure was originally developed in the USA, it is likely that the ratio of healthier and unhealthier options in the food environment is of relevance in a European context as well. Moreover, the majority of studies on the relationship between the food environment and diet have been conducted in the USA, Australia and New Zealand^(^ ^8^ ^)^, highlighting the need for studies of the food environment in a European context.

From the dietary perspective, most studies analysing the association between the food environment and diet have focused on the consumption of specific foods, for instance fruits and vegetables or fast-food meals^(^ ^6^ ^,^ ^16^ ^,^ ^22^ ^,^ ^23^ ^)^. However, an individual’s diet consists of multiple components, which may all be influenced by the food environment. As such, in the present study we used respondents’ reported consumption of different foods and drinks to derive latent (*a posteriori*) dietary patterns using principal component analysis. This is a widely used method for the analysis of dietary data that is applied to obtain data reduction by grouping highly correlated food variables into components^(^ ^24^ ^–^ ^26^ ^)^.

We aimed to test the associations of absolute and relative measures of exposure to food retailers with dietary patterns, using simpler and more complex measures. We hypothesized that: (i) more complex absolute measures, such as spatial accessibility, provide stronger associations with dietary patterns than simpler absolute measures; and (ii) relative measures of exposure to food retailers produce stronger associations with dietary patterns than when absolute measures of exposure are used.

## Methods

### Study design, sampling and participants

The current study was part of the European SPOTLIGHT project^(^ ^27^ ^)^. A web-based survey was conducted in five European urban regions: Ghent and suburbs (Belgium), Paris and inner suburbs (France), Budapest and suburbs (Hungary), the Randstad (a conurbation including the cities of Amsterdam, Rotterdam, The Hague and Utrecht in the Netherlands) and Greater London (UK). We randomly sampled twelve neighbourhoods in each urban region, based on a combination of residential density and socio-economic status (SES) data at the neighbourhood level. This resulted in four pre-specified neighbourhood types: low SES/low residential density, low SES/high residential density, high SES/low residential density and high SES/high residential density. Three neighbourhoods of each type were randomly sampled (i.e. twelve per country, sixty in total). Neighbourhoods were defined as the smallest-scale local administrative boundaries for all countries except for Hungary. The administrative boundaries for Budapest region were much larger and more heterogeneous compared with the other regions under study; therefore, to make neighbourhoods comparable across countries, we defined one square kilometre (1 km^2^) area as the study area in Hungary. Mean neighbourhood sizes ranged from 0·3 km^2^ in France to 3·6 km^2^ in the UK. Most of the participants (*n* 4942) were from Ghent (32·2 %) and the fewest participants were from the UK (10·6 %). Detailed descriptions of the neighbourhoods’ characteristics and sampling, study design and participant recruitment have been provided elsewhere^(^ ^28^ ^)^.

Between February and September 2014, individuals aged 18 years or older living in the selected neighbourhoods were invited to participate in an online survey regarding the food and physical activity environments. The questionnaire included questions on demographics, residential neighbourhood perceptions, social environmental factors, health, motivations for and barriers to engaging in healthy behaviours, dietary behaviours, and self-reported weight and height. A total of 6037 individuals participated in the study (10·8 % of the 55 893 invited). Local ethics committees in each participating country approved the study. All survey participants provided informed consent.

### Measures

#### Exposure to the food environment (independent variables)

The food environment in the selected residential neighbourhoods was objectively measured using the validated SPOTLIGHT Virtual Audit Tool (SPOTLIGHT-VAT) from February to September 2014. More information about the psychometric properties of the tool can be found elsewhere^(^ ^29^ ^)^. Briefly, the intra-observer reliability ranged from 92 % agreement (*κ* = 0·65) to 100 % agreement (*κ* = 1·00), and the inter-observer reliability ranged from 79 % agreement (*κ* = 0·44) to 99 % agreement (*κ* = 0·58). All street segments from fifty-seven residential neighbourhoods were virtually audited; three neighbourhoods were excluded from the analysis because they were not covered by Google Street View at the time of data collection or had no food retailers present. During the audit, researchers rated a total of 4482 street segments on forty-two items representing eight dimensions of the food and physical activity environments. In addition, we stored geographical coordinates as well as the type of each food outlet in a Geographical Information System.

**Fig. 1:**
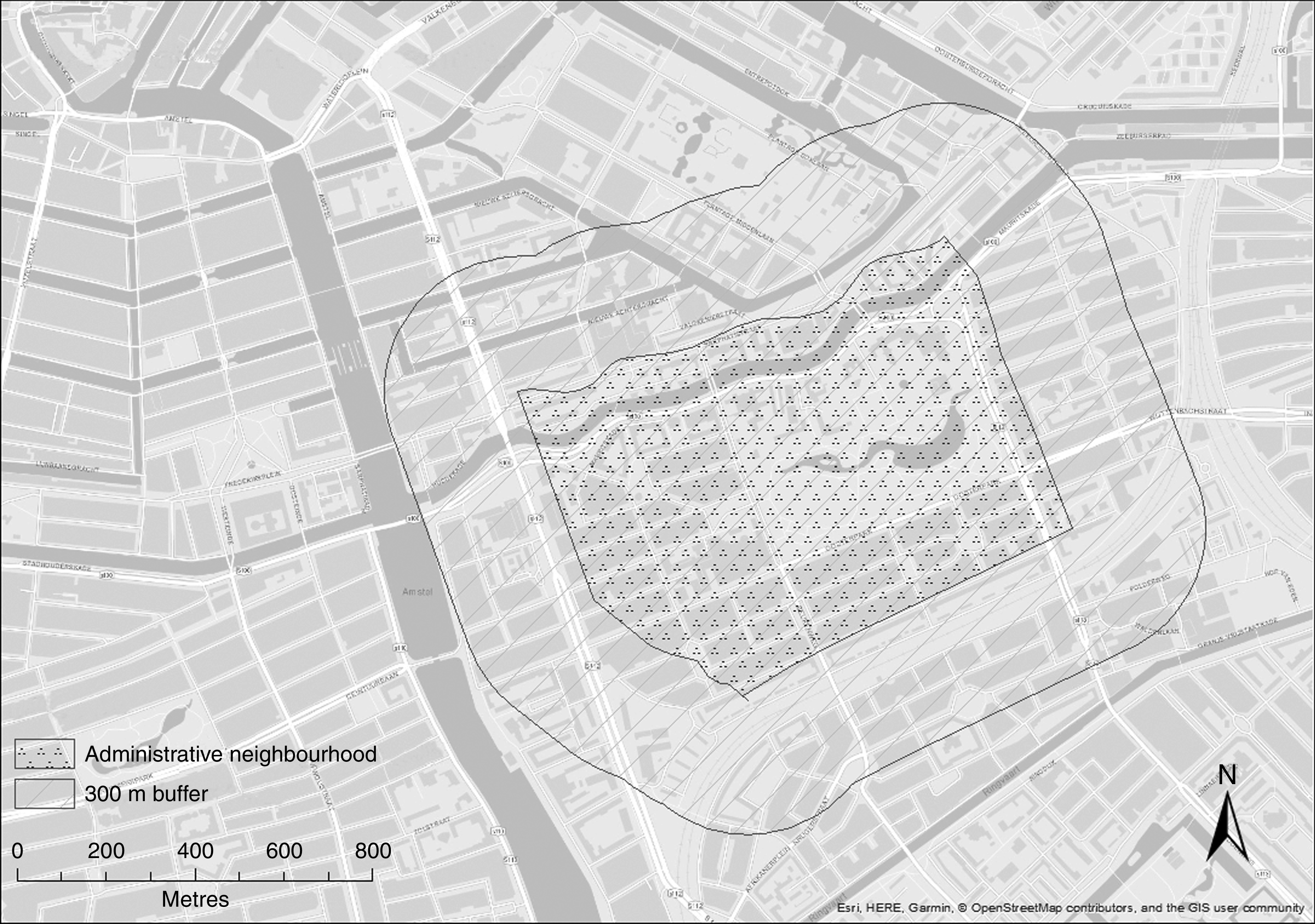
Example of how the 300m Euclidean buffer around the administrative neighbourhoods was constructed using data for Oosterparkbuurt, the Netherlands

The food retailers analysed in the present study were supermarkets, local food shops (such as butchers and bakeries), fast-food/takeaway restaurants, cafés/bars and convenience/liquor stores (this category includes convenience stores that may or may not sell alcohol and stores that sell alcohol only). Classifying food retailers according to their healthfulness is not a straightforward procedure and there is no clear definition on the healthiness of food retailers. Even though the relationship between access to supermarkets and healthier dietary habits is not fully understood, supermarkets are often considered a source of healthy foods^(^ ^30^ ^)^. The literature shows less consensus when it comes to access to restaurants: while eating away from home has been associated with lower diet quality, especially increased total energy, and both higher fat and lower micronutrient intakes^(^ ^31^ ^,^ ^32^ ^)^, full-service restaurants and fast-food restaurants might play different roles on diet and health outcomes^(^ ^33^ ^,^ ^34^ ^)^. Therefore, we considered fast-food/takeaway restaurants, cafés/bars and convenience/liquor stores as ‘food retailers providing less healthy options’. We also performed a sensitivity analysis including full-service restaurants in the latter category. We considered supermarkets and local food shops as ‘food retailers providing more healthy options’. For the sake of simplicity, in the present paper these categories are referred to as ‘less healthy food retailers’ and ‘healthier food retailers’, respectively. Using ArcGIS version 10.4, all food retailers within the limits of each individual’s residential neighbourhood (defined using administrative boundaries) plus a 300 m Euclidian buffer around it were geo-localised. We added the 300 m Euclidian buffer around the neighbourhoods to also capture a potentially relevant neighbourhood area for those living near the border of the administrative neighbourhood. An example of how this buffer around the administrative neighbourhoods was constructed is given in the Fig. 1.

To understand how different measures of exposure to the food environment were related to dietary patterns, we created six exposure variables. First, based on a common measure of exposure described in the literature^(^ ^11^ ^)^, we created two absolute density-based measures: density of healthier food retailers and density of less healthy food retailers per square kilometre. Then, we calculated two spatial access scores: spatial access to healthier and less healthy food retailers. Spatial access measures take account of both proximity and availability of food retailers. To compute this measure, the Euclidian distances from the participant’s address to each food retailer in the neighbourhood are calculated (which accounts for proximity measures). By summing the inverse distances calculated for the different food retailers in the neighbourhood, availability is also taken into account. In this way, the more food retailers are present in a neighbourhood, the more (inverse) distances will be summed. Therefore, the highest spatial access score is assigned to an individual living at the shortest distance to the highest number of food retailers. In addition, to obtain a density-based relative indicator, we calculated the mRFEI^(^ ^21^ ^)^ which represents the proportion of healthier food retailers in relation to the total amount of food retailers in the neighbourhood. Finally, to obtain a relative indicator based on proximity, density and variety, we created a ratio for the spatial access scores where individuals with a higher ratio score have a higher access to healthier food retailers relative to the total amount of food retailers in the neighbourhood. The equations used for calculating the exposure measures as well as a classification of the measures into ‘simpler absolute measures’, ‘complex absolute measures’ and ‘complex relative measures’ are presented in Table 1.

**Table 1:** Description and classification of the exposure measures used in the SPOTLIGHT project

Simpler absolute measures		
Density of healthier food retailers	=	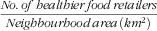
Density of less healthy food retailers	=	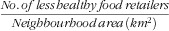
Complex absolute measures		
Spatial access to healthier food retailers	=	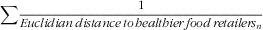
Spatial access to less healthy food retailers	=	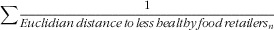
Complex relative measures		
Modified Retail Food Environment Index (mRFEI)	=	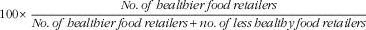
Ratio of spatial access scores	=	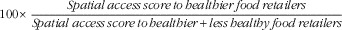

As the units of measurement across the measures used are different, and the independent variables were not normally distributed, we split the independent variables into tertiles to increase comparability.

#### Dietary patterns (outcome variables)

The weekly frequency of consumption of fruits, vegetables, fish, fast foods, sweets and sweetened beverages was assessed through the online survey by asking the following question for each of the items: ‘How many times a week do you eat fruits, vegetables, fish, […]?’ There were nine response options ranging from ‘once a week or less’ to ‘more than twice a day’.

Due to the non-normal distribution, the dietary variables were square-root-transformed. To identify common patterns of food consumption, we performed principal component analysis of the square-root-transformed variables using the correlation matrix and varimax rotation. By observing the Kaiser criterion (eigenvalue greater than 1), the scree plot and the interpretability of components, we decided to retain two principal components. Food items with an absolute factor loading greater than 0·30 were considered part of the corresponding component. The first rotated component was composed of fruits (factor loading = 0·79), vegetables (0·81) and fish (0·55); and the second rotated component was composed of sweets (0·75), sweetened beverages (0·70) and fast foods (0·40). The total variance explained by these two components was 47·6 %. Scores reflecting the weighted values of each food item in each of the respective components were then assigned to individuals^(^ ^24^ ^–^ ^26^ ^)^. The first component was named ‘healthy dietary pattern’ and the second component was named ‘less healthy dietary pattern’. These are standardized scores (mean of 0 and sd of 1). Since the scores were centred around 0, the interquartile range (IQR) for the healthy dietary pattern was −0·6 to 0·5; and the IQR for the less healthy dietary pattern was −0·8 to 0·6. These scores were multiplied by 1000 to enhance interpretation of the regression coefficients. Therefore, a 1000-point change in the outcome would represent a change equivalent to 1sd. A higher score on the ‘healthy dietary pattern’ represents a higher frequency of consumption of foods on this pattern, namely fruits, vegetables and fish. A higher score on the ‘less healthy dietary pattern’ represents a higher frequency of consumption of foods on this pattern, namely fast foods, sweets and sweetened beverages.

### Covariates

We adjusted for participants’ age, sex, educational attainment, household composition (total number of adults and children) and urban region. Educational systems differ across countries, so the educational attainment variable was categorized into two groups that were internationally comparable: ‘lower’ (secondary education or less) and ‘higher’ (college or university level) education. Household composition was categorized into three groups: ‘one adult, no child’, ‘two adults, no child’ and ‘adult(s) and child(ren)’. To minimize bias resulting from residential self-selection^(^ ^35^ ^)^, we adjusted for three variables related to neighbourhood choice. We asked participants if the presence of restaurants was a factor that influenced their decision to live in their current neighbourhood; this variable was named ‘preference for restaurants in the neighbourhood’ (no/yes). We also asked whether participants spent most of their spare time in their residential neighbourhood (no/yes) and about their duration of residency (‘less than 10 years’/‘10 years or more’). We also present a sensitivity analysis unadjusted for the self-selection variables.

### Statistical analysis

Individuals whose residential addresses were located outside the selected neighbourhoods were excluded from the analysis. We also excluded individuals living in a neighbourhood where Google Street View and geo-localisation of food retailers was not possible during the time of data collection. Individuals living in neighbourhoods without any food retailers were also excluded since it would not be possible to calculate the relative measure of exposure for them. In total 1095 participants were excluded, resulting in a final analytical sample of 4942 respondents distributed over fifty-seven neighbourhoods. We handled missing data under the assumption that data were missing at random by applying multiple imputation via predictive mean matching to all variables in the analysis, namely outcome, determinants and covariates^(^ ^36^ ^)^. As recommended by Rubin^(^ ^37^ ^)^ and Bodner^(^ ^38^ ^)^, the missing value patterns in our data set were evaluated showing that the percentage of missing values ranged from 1 % (age) to 30·2 % (preference for restaurants in the neighbourhood). For this reason, we chose to impute thirty data sets.

We performed descriptive statistics using non-imputed data. To examine the extent to which the six different measures of exposure to the food environment were unique, we calculated Pearson’s correlation coefficients. Pooled results from imputed data were used to answer our research questions. We built six different models to test the association between the six different exposure measures (density of healthier and density of less healthy food retailers; spatial access to healthier and spatial access to less healthy food retailers; mRFEI and ratio of spatial access scores) and the outcomes healthy and less healthy dietary patterns. Due to the clustered nature of our data, we performed linear models using generalized estimating equations with an exchangeable structure and with the neighbourhoods as a grouping variable^(^ ^39^ ^)^. All models were *a priori* adjusted for age, sex, educational attainment, household composition, urban region and the three variables related to neighbourhood choice. Statistical significance was determined at an *α* level of 5 %.

Descriptive and multilevel linear regression analyses, as well as the multiple imputation procedure, were performed using the statistical software package Stata^®^ version 14.1. Spatial analyses were conducted in ArcGIS version 10.4.

## Results

Table 2 describes the characteristics of participants. The median density of food retailers providing more healthy options was 1·42 per km^2^ (IQR 0·68–6·19) and with a median of 2·31 food retailers per km^2^ (IQR 0·60–9·56), the density of food retailers providing less healthy options was much higher. The mRFEI indicated that the median proportion of healthier food retailers in relation to all food retailers in the neighbourhoods was 49·49 %. The median score for spatial access to healthier food retailers was 0·009 (IQR 0·004–0·36) and for spatial access to less healthy food retailers was 0·008 (IQR 0·001–0·044). The ratio for the spatial access scores indicating the relative median proportion of healthier food retailers in the neighbourhoods was 46·35 %. The median density score for the healthier dietary pattern was −85·9 (IQR −591·1, 510·6) and for the less healthy dietary pattern was −63·6 (IQR −767·6, 583·1; data not shown).

**Table 2:** Descriptive characteristics of the participants: adults in neighbourhoods from five urban regions in Europe, February–September 2014. The SPOTLIGHT project (*n* 4942)

	*n*	Mean, % or median	sd or IQR
Age (years), mean and sd	4893	52·3	16·4
Sex (%)	4893		
Female		55·5	
Educational attainment (%)	4470		
Higher		54·1	
Household composition (%)	4471		
1 adult, no children		22·4	
2 adults, no children		47·8	
Adult(s) with child(ren)		29·8	
Urban regions (%)	4942		
Ghent and suburbs (Belgium)		34·2	
Paris and suburbs (France)		14·3	
Budapest and suburbs (Hungary)		14·4	
The Randstad (the Netherlands)		26·6	
Greater London (the UK)		10·6	
Duration of residency in this neighbourhood (%)	4741		
Less than 10 years		35·0	
10 years or more		65·0	
Spare time spent in the neighbourhood (%)	4801		
Yes		71·8	
Preference for restaurants in the neighbourhood (%)	3449		
Yes		16·2	
Density of healthier food retailers, median and IQR*	4942	1·42	0·68–6·19
Density of less healthy food retailers, median and IQR*	4942	2·31	0·60–9·56
mRFEI, median and IQR†	4942	49·49	32·50–62·50
Spatial access to healthier food retailers, median and IQR‡	4942	0·009	0·004–0·036
Spatial access to less healthy food retailers, median and IQR‡	4942	0·008	0·001–0·044
Ratio for the spatial access scores, median and IQR§	4942	46·35	28·69–84·61
Score for healthy dietary pattern, mean and sd ║	3950	0	1000
Score for less healthy dietary pattern, mean and sd ║	3950	0	1000

IQR, interquartile range; mRFEI, modified Retail Food Environment Index.

* Density represents the count of food retailers divided by the neighbourhood area in square kilometres.

† mRFEI represents the proportion of healthier food retailers in relation to the total number of food retailers in the neighbourhood.

‡ Spatial access score represents an inverse function of the sum of the calculated distances from individuals’ home address to each healthier and less healthy food outlet in the residential neighbourhood.

§ Ratio for the spatial access scores represents spatial access scores to healthier food retailers divided by healthier plus less healthy food retailers.

║ Scores for healthier and less healthy dietary patterns were multiplied by 1000.

Table 3 shows the Pearson correlation coefficients for the six exposure measures. The density of healthier food retailers was strongly associated with the density of less healthy food retailers (*r* = 0·79). Spatial access to healthier food retailers was strongly associated with the density of both healthier (*r* = 0·84) and less healthy (*r* = 0·75) food retailers, while this was not the case for the densities and spatial access to less healthy food retailers (*r* = 0·15 and *r* = 0·26, respectively). Differently from the density measures, the two spatial access measures were not strongly associated (*r* = 0·16). While the ratio of spatial access was strongly associated to the mRFEI (*r* = 0·87), it was not strongly associated to the other four absolute exposure measures.

**Table 3:** Pearson correlation coefficients for the six measures of exposure to the food environment among adults in neighbourhoods from five urban regions in Europe, February–September 2014. The SPOTLIGHT project (*n* 4942)

	Density of healthier food retailers	Density of less healthy food retailers	mRFEI	Spatial access to healthier food retailers	Spatial access to less healthy food retailers	Ratio of spatial access scores
Density of healthier food retailers	1					
Density of less healthy food retailers	0·7884	1				
mRFEI	−0·0486	−0·2999	1			
Spatial access to healthier food retailers	0·8418	0·7475	−0·0952	1		
Spatial access to less healthy food retailers	0·1472	0·2551	−0·0879	0·1649	1	
Ratio of spatial access scores	0·0290	−0·2376	0·8720	0·0368	−0·1432	1

mRFEI, modified Retail Food Environment Index.

Table 4 shows the associations between the absolute measures of exposure and both healthy and less healthy dietary patterns. The highest density of less healthy food retailers was significantly associated with a lower score on the less healthy dietary pattern. The effect size, however, is rather small considering that the outcome measure was multiplied by 1000 to enhance interpretation of the results (*β* = −129·6; 95 % CI −224·3, −34·8).

**Table 4:** Coefficients and 95 % CI as derived from generalized estimating equation–linear regression analyses indicating the associations of absolute measures of exposure to food retailers with dietary patterns among adults in neighbourhoods from five urban regions in Europe, February–September 2014. The SPOTLIGHT Project (*n* 4942)

	Healthy dietary pattern* †			Less healthy dietary pattern† ‡		
	Coefficient	95 % CI	*P* value	Coefficient	95 % CI	*P* value
Tertiles of density of healthier food retailers per km^2^ §						
Lowest	1			1		
Medium	−48·2	−233·6, 137·3	0·611	13·7	−98·1, 125·5	0·810
Highest	−10·5	−178·8, 157·8	0·903	−56·6	−163·0, 49·9	0·297
Tertiles of density of less healthy food retailers per km^2^ ║						
Lowest	1			1		
Medium	−78·1	−244·9, 88·8	0·359	−61·3	−176·3, 53·7	0·296
Highest	−23·3	−181·3, 122·7	0·706	−129·6	−224·3, −34·8	0·007
Tertiles of spatial access score for healthier food retailers§						
Lowest	1			1		
Medium	−77·2	−183·1, 28·7	0·153	−18·2	−115·5, 79·2	0·714
Highest	−99·7	−222·9, 23·5	0·113	−34·4	−133·8, 65·0	0·498
Tertiles of spatial access score for less healthy food retailers║						
Lowest	1			1		
Medium	−68·0	−164·9, 28·9	0·169	−6·67	−90·9, 77·6	0·876
Highest	−100·0	−224·3, 24·2	0·114	−9·00	−117·4, 99·4	0·871

Dietary patterns were obtained from principal component analysis. All models were adjusted for age, sex, educational attainment, household composition, urban region and self-selection variables.

* Healthy dietary pattern is composed of fruits, vegetables and fish.

† Scores for healthy and less healthy dietary patterns were multiplied by 1000.

‡ Less healthy dietary pattern is composed of fast foods, sweets and sweetened beverages.

§ Healthier food retailers: supermarkets and local shops;

║ Less healthy food retailers: fast-food restaurants, cafés/bars and convenience/liquor stores.

Table 5 shows the associations between the relative measures of exposure and the dietary patterns. We did not find any significant association with the dietary patterns using either of the relative measures tested, and the effect sizes are negligible.

**Table 5:** Coefficients and 95 % CI as derived from generalized estimating equation–linear regression analyses indicating the associations of relative measures of exposure to food retailers with dietary patterns among adults in neighbourhoods from five urban regions in Europe, February–September 2014. The SPOTLIGHT Project (*n* 4942)

	Healthy dietary pattern* †			Less healthy dietary pattern† ‡		
	Coefficient	95 % CI	*P* value	Coefficient	95 % CI	*P* value
Tertiles of the mRFEI						
Lowest	1			1		
Medium	−28·4	−231·2, 174·4	0·784	13·1	−107·2, 133·4	0·831
Highest	86·8	−88·7, 262·4	0·332	93·4	−9·0, 195·7	0·074
Tertiles of the ratio for spatial access scores						
Lowest	1			1		
Medium	−58·8	−173·5, 67·9	0·391	20·4	−69·6, 110·4	0·657
Highest	48·0	−94·5, 190·5	0·509	36·0	−65·0, 137·1	0·484

mRFEI, modified Retail Food Environment Index. Dietary patterns were obtained from principal component analysis. All models were adjusted for age, sex, educational attainment, household composition, urban region and self-selection variables.

* Healthy dietary pattern is composed of fruits, vegetables and fish.

† Scores for healthy and less healthy dietary patterns were multiplied by 1000.

‡ Less healthy dietary pattern is composed of fast foods, sweets and sweetened beverages.

The online supplementary material, Supplementary Tables 1 and 2, shows the results from sensitivity analyses where we included full-service restaurants in the category of less healthy food retailers. These results are highly comparable to the main analyses where full-service restaurants were left out. In Supplementary Table 1, the most notable difference is that the association between density of less healthy food retailers and less healthy dietary patterns was no longer significant when full-service restaurants were included in the less healthy category. In addition, although still not statistically significant and with very small effect sizes, the direction of the association between spatial access to less healthy food retailers and the less healthy dietary pattern changed: the previously negative association became positive when full-service restaurants were considered. In contrast, in Supplemental Table 2, which shows the association for relative measures, the previously positive association between the ratio for spatial access scores and less healthy dietary patterns became negative when full-service restaurants were included in the less healthy food retailers. The effect sizes, in turn, became even smaller. Supplemental Tables 3 and 4 show the results from sensitivity analyses where we did not adjust our models for the self-selection variables. Results from these analyses are comparable to the main analyses which are adjusted for self-selection.

## Discussion

We tested the association of different neighbourhood food exposure measures with dietary patterns. We aimed to provide a comprehensive picture of these potential associations by using both simpler and more complex measures of exposure. We used density and spatial access scores (with the latter accounting for both density and proximity) and also relative measures of exposure to food retailers, namely the mRFEI and a ratio of spatial access scores to healthier and less healthy food retailers.

The correlation analyses showed important differences across the exposure measures used. The fact that density of healthier food retailers was strongly correlated with the density of less healthy food retailers suggests that healthier and less healthy food retailers often co-locate^(^ ^40^ ^)^. Spatial access to healthier food retailers, but not spatial access to less healthy food retailers, was associated with both densities. The spatial access score is a more tailored measure than only density because in its calculation the weighted distances to food retailers are derived and summed up – which then accounts for both density and proximity. However, when there are few food retailers in the neighbourhood, the spatial access measure will be very similar to the density measure, as only a few distances will be added in the calculation. Therefore, because in the present study there was on average a higher number of less healthy food retailers than healthier ones, the spatial access to less healthy food retailers was not strongly related to either of the two density measures. This may also explain why the two spatial access measures were not strongly correlated. Not surprisingly, the ratio of spatial access was strongly correlated to the mRFEI, as both are relative measures accounting for the ratio of healthier food retailers to the total amount of food retailers in a neighbourhood. On the other hand, the two ratio measures were not strongly correlated to the four absolute exposure measures, confirming that absolute and relative measures assess different aspects of the food environment.

The density of less healthy food retailers was associated with lower scores on the less healthy dietary pattern. This negative association is unexpected. However, it is interesting to note that all associations from Table 3, which presents the associations using the absolute measures of exposure, are related to lower scores on any of the dietary patterns – although effect sizes are all very small and non-significant. In contrast, in Table 4, which presents the associations using the relative measures of exposures, most measures of exposure are related to higher scores on both dietary patterns. Considering the lack of associations and the very small effect sizes, we cannot confirm our hypothesis that more complex and relative exposure measures would be more consistently related to dietary patterns than simpler measures. Nevertheless, the observed pattern with the direction of the associations across Tables 3 and 4 reinforces the conclusion obtained from the correlation analysis (Table 2) on the different nature of absolute and relative measures of exposure.

We could not demonstrate that relative measures of exposure could provide more consistent associations between the food environment and diet, yet there has been a growing body of literature indicating that^(^ ^16^ ^,^ ^41^ ^,^ ^42^ ^)^. There is evidence suggesting that despite the presence of less healthy food retailers in the neighbourhood might encourage the consumption of unhealthier foods, the concomitant presence of healthier options may reduce the potential harmful effect on individuals’ diets^(^ ^16^ ^)^. However, as our results indicate, at best, that the choice of exposure measure has an impact on the findings, the reasons for the adjustment for the broader food environment using relative measures (that typically account for the presence of both healthy and less healthy food retailers) should be made explicit and discussed. For instance, it may be that adjustment for the broader food environment is only relevant when the food retailers considered are of a similar type, for example healthier and less healthy dining options or healthier and less healthy food stores. On this matter, Polsky *et al.* found that a higher presence of fast-food restaurants relative to the presence of other restaurant types, including full-service, was associated with higher odds of obesity. These results did not change when in a sensitivity analysis they adjusted their models for the presence of healthier food options such as supermarkets and fruit and vegetable shops^(^ ^20^ ^)^.

Although more sophisticated measures of exposure might, theoretically, get closer to better representing the residential food environment – with stronger correlations to dietary outcomes – this was not confirmed by the present study. A study using a similar complex measure of exposure to test the association between spatial access to fast-food outlets and weight status also did not find significant associations^(^ ^13^ ^)^. Even though we used more comprehensive measures of exposure, given all the other factors that influence the relationship between the environment and behaviour, we may not have been able to capture the complexity of the relationships by which the food environment influences the dietary patterns of individuals. The difficulty of representing exposure to the food environment and its association with health behaviours has been reported before^(^ ^8^ ^,^ ^23^ ^,^ ^43^ ^)^. It has been proposed that different neighbourhood definitions and multiple neighbourhood contexts (e.g. social, food and physical activity environment) should be taken into account while trying to model how exposure to the environment might influence health outcomes^(^ ^44^ ^)^. However, there is no consensus in the literature on what the best exposure measure would be and due to the particularities of each research setting, reaching consensus may not be possible or even appropriate. Nevertheless, the researchers’ attempt on working towards the best possible representation of the food environment, within each reality, and ways to better characterize individual exposure, should always be reported and acknowledged^(^ ^43^ ^)^.

Some limitations of the current study need to be addressed. First, the dietary pattern measures were based on a series of basic food frequency questions, and this measure may not have been sufficiently sensitive to detect all meaningful associations. The use of crude dietary data has been reported as a common limitation and one of the potential explanations for the inconsistency found in food environment research^(^ ^45^ ^)^. Another potential limitation of the present study, as well as most of the previous studies, is that by focusing on the food environment around individuals’ homes, we may have failed to include other relevant food environments, such as those in and around work or leisure locations. Dietary habits might be influenced by individuals’ ethnic and socio-economic background. Unfortunately, due to ethical constrains, we were not able to collect information on ethnicity and income in two countries in our study. We have adjusted our models for relevant individual- and neighbourhood-level variables, including education, household composition and self-selection. We sampled the neighbourhoods based on neighbourhood SES and density and we performed adjustment for country. However, as in most observational studies, some level of residual confounding might have occurred. Strengths of the present study include the fact that it contributes to further explorations of differences between relative and absolute exposure measures of the food environment and the fact that we used a comprehensive statistical approach guided by explicit hypotheses; we accounted for the clustered nature of our data and sought to overcome potential bias due to missing data by performing multiple imputation. Finally, our large sample size, collected from five European countries, contributes to the external validity of our findings. Even though the low response rate might have produced a selective sample, the distribution of participants was well balanced in terms of sociodemographic characteristics such as percentage of males and females, level of education and BMI. Therefore, although it is not possible to exclude the possibility of residual biases within this sample, this is an indication that the sample is broadly representative of the population regarding these characteristics.

## Conclusions

We examined different measures of exposure to explore the broader residential food environment and its association with dietary patterns. We could not confirm our hypotheses that more complex absolute measures or relative measures of exposure to food retailers would produce stronger associations with dietary patterns. We had some indication that absolute and relative measures of exposure assess different aspects of the food environment and this might be reflected in the direction of associations. However, given the lack of significant findings, this needs to be further explored.
